# Projected Impacts of Climate Change on the Range Expansion of the Invasive Straggler Daisy (*Calyptocarpus vialis*) in the Northwestern Indian Himalayan Region

**DOI:** 10.3390/plants13010068

**Published:** 2023-12-25

**Authors:** Roop Lal, Saurav Chauhan, Amarpreet Kaur, Vikrant Jaryan, Ravinder K. Kohli, Rishikesh Singh, Harminder P. Singh, Shalinder Kaur, Daizy R. Batish

**Affiliations:** 1 Department of Botany, Panjab University, Chandigarh 160014, India; 2 Faculty of Basic Sciences, Shoolini University of Biotechnology and Management Sciences, Solan 173229, Himachal Pradesh, India; 3 Department of Life Sciences, Allied Health Sciences & Agriculture Sciences, Sant Baba Bhag Singh University, Village Khiala, Padhiana, Jalandhar 144030, Punjab, India; 4 Amity University Punjab, Mohali 140306, Punjab, India; 5 Amity School of Earth and Environment Sciences, Amity University Punjab, Mohali 140306, Punjab, India; 6 Department of Environment Studies, Panjab University, Chandigarh 160014, India

**Keywords:** bioclimatic factors, climate change, MaxEnt, northwestern Indian Himalayan region, receiver operating characteristic (ROC), topographic factors

## Abstract

Human-induced climate change modifies plant species distribution, reorganizing ecologically suitable habitats for invasive species. In this study, we identified the environmental factors that are important for the spread of *Calyptocarpus vialis*, an emerging invasive weed in the northwestern Indian Himalayan Region (IHR), along with possible habitats of the weed under current climatic scenarios and potential range expansion under several representative concentration pathways (RCPs) using MaxEnt niche modeling. The prediction had a high AUC (area under the curve) value of 0.894 ± 0.010 and a remarkable correlation between the test and expected omission rates. BIO15 (precipitation seasonality; 38.8%) and BIO1 (annual mean temperature; 35.7%) had the greatest impact on the probable distribution of *C. vialis*, followed by elevation (11.7%) and landcover (6.3%). The findings show that, unlike the current situation, “high” and “very high” suitability areas would rise while less-suited habitats would disappear. All RCPs (2.6, 4.5, 6.0, and 8.5) indicate the expansion of *C. vialis* in “high” suitability areas, but RCP 4.5 predicts contraction, and RCPs 2.6, 6.0, and 8.5 predict expansion in “very high” probability areas. The current distribution of *C. vialis* is 21.59% of the total area of the state, with “medium” to “high” invasion suitability, but under the RCP 8.5 scenario, it might grow by 10% by 2070. The study also reveals that *C. vialis* may expand its niche at both lower and higher elevations. This study clarifies how bioclimatic and topographic factors affect the dispersion of invasive species in the biodiverse IHR. Policymakers and land-use managers can utilize the data to monitor *C. vialis* hotspots and develop scientifically sound management methods.

## 1. Introduction

Human-driven modifications of climate strongly transform the potential matrix of plant species distribution by reshuffling ecologically suitable habitats [1]. Changes in climate have led to increased temperatures, erratic precipitation, enhanced atmospheric carbon dioxide, unpredictable seasonal shifts, extreme weather events, prolonged drought periods, frequent forest fires, acidification of water bodies, desertification of drylands, the formation of heat islands, and habitat loss and fragmentation [2,3]. In addition to alterations in vegetation patterns and community structure and function, climate change has enhanced biotic invasions, thereby disrupting ecosystems’ dynamics, productivity, resistance, and resilience [4,5,6].

Plant invasions induced by global climate change are one of the most prominent challenges in community ecology due to their massive economic and ecological consequences [7]. Since most of the invasive plant species are opportunistic generalists with wide ecological amplitude [4,8], these can better adapt to the changing environmental conditions compared with the natives [9]. These invasive species have a strong impact on fragile and vulnerable ecosystems such as mountain regions [10], island and coastal zones, and protected areas [6]. Invasive plant species acquire a competitive edge over the natives owing to their exceptional traits such as strong reproductive and dispersal strategies, tolerance to abiotic and biotic stresses, plastic and adaptive responses, efficient resource capture, and phytotoxic potential [11,12,13,14,15].

Comprehending the distribution range and patterns of invasive plant species is difficult, and predicting their potential spread is even more challenging. Of late, studies have focused on computational models for estimating the likelihood of invasive species’ spatio-temporal spread using current knowledge of their distribution and different environmental variables [16,17]. Ecological niche modeling (ENM) predicts a species’ potential spread based on species presence and absence data, which helps in determining the strength of the relationship between a species and its environment [18,19]. MaxEnt is one such comprehensive ENM tool commonly employed for predicting species distribution by conservation practitioners and researchers [20,21,22]. It is a maximum entropy-based program that establishes a relationship between the environmental factors in the region where a species is found and the environmental factors of interest. Therefore, MaxEnt does not require species absence data and exploits only the predictor variables and species presence data to predict the potential species’ distribution [16,22,23]. Consequently, it is often more beneficial compared with the presence- or absence-based modeling methods. Currently, MaxEnt is commonly used for envisaging the possible habitat expansion of invasive species in relation to global environmental changes [24,25,26,27]. Such investigations have indicated that the influence of changing climates increases the likelihood of invasion [28].

Several plant species have been reported to be invasive in the ecologically sensitive and fragile ecosystems of the northwestern Indian Himalayan Region (IHR). However, to date, studies investigating the potential niche expansion of invasive species along the elevation gradient in the region are limited. It is important to employ computational models to estimate the potential spread of invasive species in the Himalayan belt, as research in this direction will facilitate conservation efforts and help in interpreting the connection among bioclimatic and topographic variables and the probable distribution of prominent invaders in the region. This is of particular significance when considering newly introduced invasive species, as they may currently exhibit a limited geographical spread, yet they harbor the potential for significant future expansion. An example of this is *Calyptocarpus vialis* Less. (=*Synedrella vialis*; straggler daisy, horseherb, creeping Cinderella weed; Asteraceae), an emerging invasive weed in the lower Shivalik region of the Indian Himalayas [29]. It is native to eastern Mexico, South Central Texas, and the West Indies and it has been designated as a rapidly spreading invasive weed in tropical and subtropical areas worldwide [30,31,32]. It is also distributed in many Indian states like Uttar Pradesh [33], Karnataka [34], Himachal Pradesh [35], Punjab [36], and Kerala [37]. *C. vialis* is found along roadsides, in open or shady places with 40–60% moisture content, and in ruderal habitats (personal observations). Rapid prostrate growth of plants results in the formation of carpet-like patches in the invaded habitats. High seed output, phytotoxic potential, active reproduction via both sexual and vegetative means, and wide ecological amplitude are the major invasion strategies employed by the weed during its invasion [29,37]. *C. vialis* has the capacity to extend its distribution to higher altitudes within the Himalayan belt, a region expected to undergo significant ecological shifts due to the impacts of global warming [38].

The present study investigated the spread of *C. vialis* in the northwestern IHR under a changing climate scenario. The objectives of the current study were to (a) characterize the ecological niche of *C. vialis* in the northwestern IHR and identify environmental variables important for its distribution; and (b) identify the potentially suitable habitats and niche expansion of the species in the study area by 2070, using four representative concentration pathways (RCP; 2.6, 4.5, 6.0, 8.5).

## 2. Materials and Methods

### 2.1. Study Area

**Figure 1:**
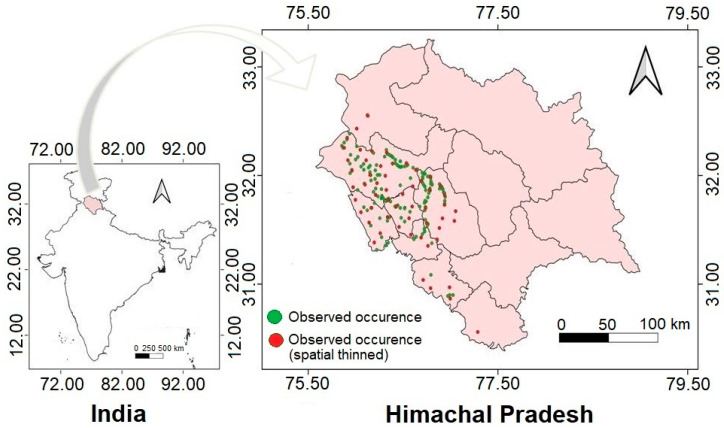
Map of the study area and the occurrence records of *Calyptocarpus vialis* used for MaxEnt modeling.

The study was undertaken in Himachal Pradesh, a northwestern Indian state, located in the Himalayan range within 30°22′40″–33°12′40″ N and 75°45′55″–79°04′20″ E (Figure 1). The state covers an area of 55,673 km^2^ and has an altitudinal gradient of 250 to 7000 m asl [39]. It has twelve districts experiencing tropical to alpine conditions (https://www.himachalworld.com, accessed on 5 December 2023). The minimum temperature at the lower altitudes (500–1000 m asl) varies between 4 and 6 °C, and at the higher altitudes (>4000 m asl), it ranges from −28 °C to −25 °C. On the other hand, the maximum temperature ranges between 38 and 42 °C at the lower altitudes and 25–28 °C at the higher altitudes [40]. Precipitation occurs in the form of rainfall and snow, with the maximum rainfall being recorded during the monsoon season and the maximum snowfall during the winter season. In 2022, the state witnessed variability in rainfall ranging from 5.7 mm (in March) to 263.4 mm (in July) with a total annual rainfall of 1086.4 mm (https://mausam.imd/gov.in.shimla/mcdata/cli_hp.pdf; accessed on 5 December 2023) and an annual snowfall varying from 25 to 204 cm (https://imdpune.gov.in/library/public/Climate%20of%20Himachal%20Pradesh.pdf; accessed on 5 December 2023).

Due to its wide altitudinal range and diverse climatic and soil types, the region supports a variety of habitats and vegetation [41]. As per the India State of Forest Report (ISFR), the recorded forest area in the state is 68.6% of the total geographical area (https://fsi.nic.in/forest-report-2021-details/; accessed on 5 December 2023). Also, 497 exotic species belonging to 85 families have been found in the state [39], which include some prominent invaders such as *Ageratum conyzoides* L., *Ageratina adenophora* (Spreng.) King & H.Rob., *Lantana camara* L., and *Parthenium hysterophorus* L. Several other exotic species, such as *Bidens pilosa* L. and *C. vialis*, have also been spreading in the region at an alarming rate [29,42]. Most of these invasive species are initially established at lower elevations and then swiftly expanded towards the higher ranges [40]. In the present study, the current habitat and potential habitat expansion of *C. vialis* are traced in the study area by applying ENM on the occurrence data collected via field surveys.

### 2.2. Data Collection

To collect occurrence data for *C. vialis*, field surveys were undertaken in the study area, covering eight districts (regions) of Bilaspur, Hamirpur, Kangra, Mandi, Shimla, Solan, Sirmour, and Una, from July to October of 2016–2020. The selection of the districts was based on personal observations and literature reporting the dominance of *C. vialis* in Himachal Pradesh [35]. The occurrence points were recorded using a Garmin eTrex Vista GPS handset. A total of 196 location points were recorded, which indicated the presence of *C. vialis* in different districts (Figure 1). The collected data were processed using suitable analytical techniques.

### 2.3. Data Processing and Predictor Variables

To ensure an even spread of the randomized dataset and avoid overprediction, the sampling bias was eliminated by spatially thinning the occurrence data with the help of the SpThin package applied to R version 3.6.3 [43]. The thinning was performed using the thin distance set of 10 km, which reduced the data points to 56. These points were used for modeling the current distribution of *C. vialis* (Figure 1).

For estimating the probable distribution of *C. vialis*, 23 variables were selected [44,45]. Of these, the nineteen Bioclimatic layers (Bio1 to Bio19) were downloaded for the period 1970–2000 from WorldClim version 2 (http://worldclim.org; accessed on 5 December 2023) and transformed from GeoTIFF to ASCII format with the help of QGIS. The Earth Explorer (https://earthexplorer.usgs.gov; accessed on 5 December 2023) was used for extracting the Shuttle Radar Topographic Mission (SRTM) digital elevation model (DEM), which was used to obtain slope and aspect data. Global 300 m landcover data and GlobCover 2009 were extracted from http://due.esrin.esa.int (accessed on 5 December 2023).

To forecast the future distribution of *C. vialis*, we chose four RCP scenarios as the estimator of radiative forcing, which is defined as perturbation in the energy balance of the earth caused by human activities, particularly the emission of greenhouse gases. In the first scenario (RCP 2.6), we envisioned a scenario characterized by minimal greenhouse gas emissions and mitigation measures, reaching a peak in radiative forcing at around 3 W/m^2^ (~490 ppm CO_2_ eq) before 2100, followed by a subsequent decline. RCP 4.5 represented a scenario of moderate to low mitigation, with a very low baseline and stabilization that would reach 4.5 W/m^2^ (~650 ppm CO_2_ eq) after 2100 without overshooting. RCP 6.0 illustrated a high-mitigation scenario with a moderate baseline and stabilization at 6 W/m^2^ (~850 ppm CO_2_ eq) after 2100 without overshooting. Lastly, RCP 8.5 signified a scenario of high baseline emissions, leading to a trajectory of rising radiative forcing peaking at 8.5 W/m^2^ (~1370 ppm CO_2_ eq) by 2100 [46]. Data for these scenarios were sourced from WorldClim for the period 2060–2080. The period was chosen to capture an intermediate stage where radiative forces are at moderate levels, thus avoiding any under- or over-prediction. This timeframe allows a comparatively accurate prediction of climate change while also offering robust long-term planning and adaptive management. Therefore, the period provides suitable insights into the potential distribution shifts in *C. vialis* over an extended period with appropriate accuracy.

The variables adhered to the shapefile of Himachal Pradesh, which was extracted from the Database of Global Administrative Areas (https://gadm.org/download_country_v3.html; accessed on 5 December 2023). To match the spatial resolution of the bioclimatic layers (30 arc s), topographic data (elevation, slope, aspect, and landcover) were resampled using the bilinear interpolation technique. The selected bioclimatic and topographic parameters were tested for multicollinearity using Pearson’s correlation, and the parameters with a very strong correlation coefficient (with a value ≥ ±0.75) were excluded from further evaluation for better interpretation and generalization [47]. The processed data were then used to predict habitat suitability using ENM.

### 2.4. Ecological Niche Modeling Algorithm

MaxEnt (version 3.4.1) was downloaded from http://biodiversityinformatics.amnh.org/open_source/maxent/ (accessed on 5 December 2023). The subsampling method was used to process the data and select parameters with 10 replications and 5000 iterations. A random test percentage was fixed at 30%, implying that 30% of the data points were chosen at random and 70% were from the training dataset [48]. The background points were set at a maximum of 10,000. Both linear (which models the mean values of the parameters) and quadratic (models the variances of the parameters) functions were adjusted, while others were set at default.

### 2.5. Model Evaluation

The area under the ROC curve (AUC) is a threshold-independent parameter, ranging from 0 to 1, employed extensively to evaluate the strength and precision of the species distribution model. Its value is calculated by plotting the fraction of accurate predictions of species presence (sensitivity) against the fraction of inaccurate predictions of species absence (1 − specificity). Since sensitivity and specificity include all the correct and incorrect presence and absence datasets, both are incorporated into the model. Further subtraction of specificity from 1 allows these metrics to proceed in the same direction [19,49]. A model with the most accurate prediction generates a curve proximal to the left axis and towards the topmost direction, whereas a model with random prediction will chase the 1:1 line [47]. Thus, the accuracy of the generated models was assessed based on the AUC value. A value less than 0.5 indicates the worst performance, whereas values greater than 0.5 reflect different levels of performance of the model as compared to a random chance. The AUC values between 0.5 and 0.6, 0.6 and 0.7, 0.7 and 0.8, 0.8 and 0.9, and 0.9 and 1.0 imply failed, poor, fair, good, and excellent performance of the model, respectively.

The comparative significance of the parameters used in the model was appraised via the jackknife procedure. Based on a logistic threshold of 10 percentile training presence, the output of MaxEnt directly predicts whether an area is good for a certain species on a scale from 0 to 1. Accordingly, the suitable habitats were classified into different categories, with values in the range of 0.0–0.10 representing “very low” suitability, 0.10–0.30 representing “low” suitability, 0.30–0.50 representing “medium” suitability, 0.50–0.70 representing “high” suitability, and 0.70–1 representing “very high” suitability [50]. The pixels in the cloglog output format show different categories of habitat suitability, which were extracted, counted, and changed into a unit of area (km^2^) to estimate the exact proportion of the region occupied by the species. The results are presented in different formats, showing the sensitivity, validity of the model, and suitability of the habitat.

### 2.6. Map Projection and Data Presentation

The maps were prepared on QGIS and projected to EPSG 4326–WGS 84. MaxEnt output is presented in three different formats: cumulative output, cloglog output, and logistic output. The jackknife test is presented as cumulative output, whereas the response curves of individual responsive variables selected after the correlation are presented as cloglog output. The Jackknife test is a tool used to ensure consistent and accurate prediction of the model even in the absence of certain variables, thereby enhancing the overall validity of the model. It identifies the environmental variables influencing the distribution of species [51,52,53]. On the other hand, the response curve indicates species responses to specific environmental variables and helps identify a range of variables where species are more likely to thrive. It also predicts the suitability of the habitat [51,53]. The current habitat suitability map and potential habitat suitability maps with different RCP scenarios are presented as logistic output.

## 3. Results

### 3.1. Model Performance and Accuracy

**Figure 2:**
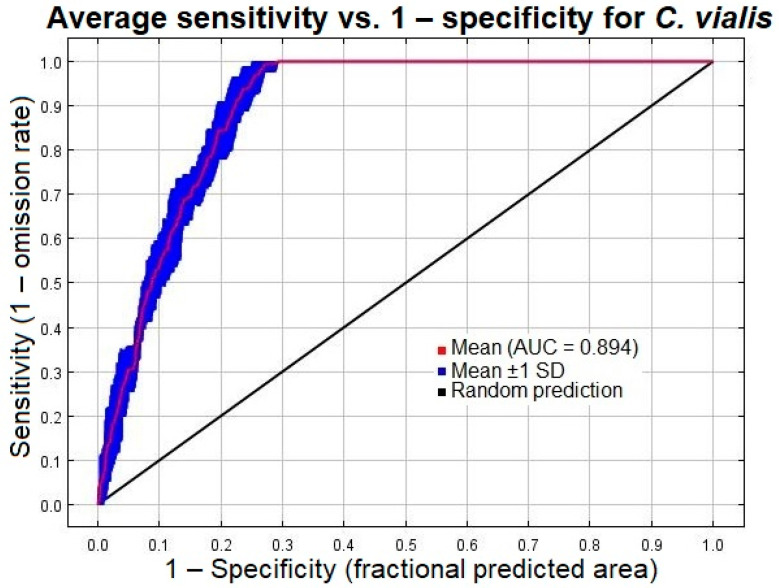
The area under the receiver operating characteristics curve for *Calyptocarpus vialis* obtained via MaxEnt modeling. Red, blue, and black curves correspond to the training, test, and random prediction data, respectively.

**Figure 3:**
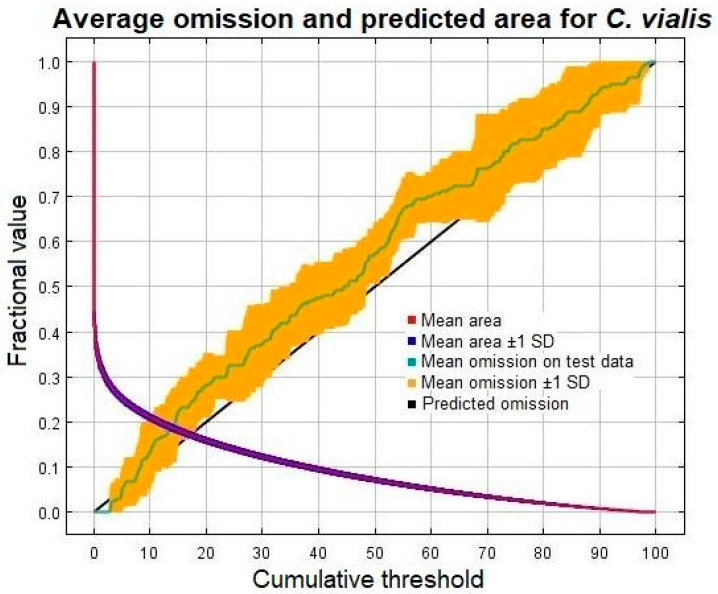
Omission analysis and predicted area for *Calyptocarpus vialis* interpreted using MaxEnt modeling.

The performance of the threshold-independent ROC curve was investigated as per the AUC values. Since the curve was in proximity to the left axis, alienated towards the upper side, aligned away from the 1:1 line, and had an AUC value of 0.894 ± 0.010, it is considered highly accurate and acceptable (Figure 2). The result suggests that the selected environmental parameters used for the calibration of the model quite accurately forecasted the distribution of *C. vialis* in the study area. In addition to ROC, the model’s validity and performance were assessed by examining the omission/commission rate (cumulative output), in which the omission (calculated on training presence as well as test records) indicates the proportions of unsuitable localities or pixels, while the predicted area represents suitable pixels. The proximity between the test and predicted omission rates further defined its accuracy (Figure 3).

### 3.2. Key Environmental Variables

**Figure 4:**
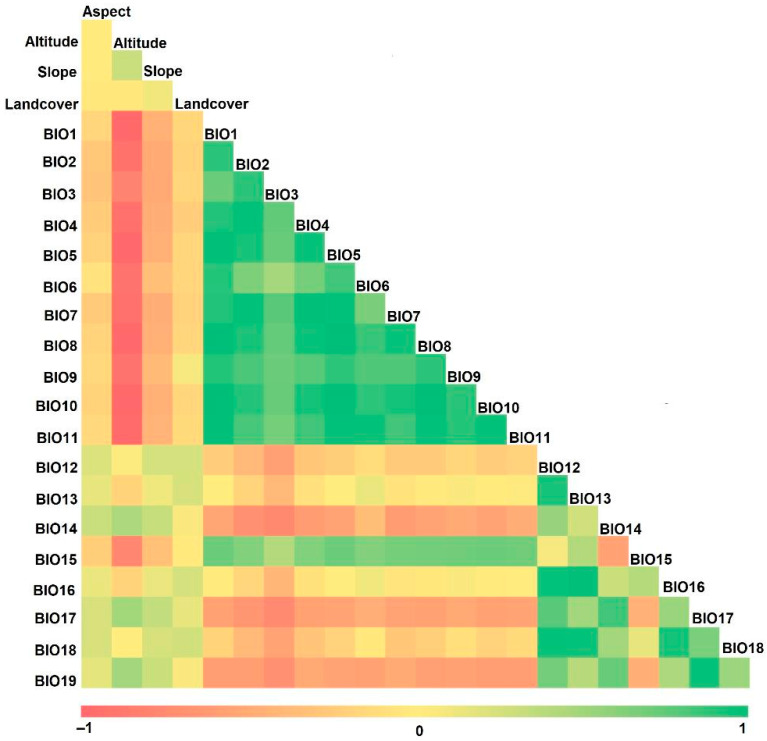
The interrelationship among selected bioclimatic and topographic parameters used in the prediction of the potential spread of *Calyptocarpus vialis*.

We did not use the Pearson multicollinearity test for 16 environmental variables because their cross-correlation values were beyond ± 0.75 (Figure 4). These variables were mean diurnal range (BIO2), isothermality (BIO3), temperature seasonality (BIO4), maximum temperature of warmest month (BIO5), minimum temperature of coldest month (BIO6), temperature annual range (BIO7), precipitation of wettest and driest month (BIO13–BIO14), mean temperature (BIO8–BIO11), and precipitation (BIO16–BIO19) of wettest, driest, warmest, and coldest quarter. The main variables that were selected after the result of the multi-collinearity test were BIO1, BIO12, BIO15, landcover, elevation, slope gradient, and slope cover (Table 1). The variable contribution analysis revealed that the spread of *C. vialis* is primarily shaped by BIO15 (38.8%), followed by BIO1 (35.7%), elevation (11.7%), landcover (6.3%), slope gradient (5%), BIO12 (1.6%), and slope aspect (1%) (Table 1).

**Figure 5:**
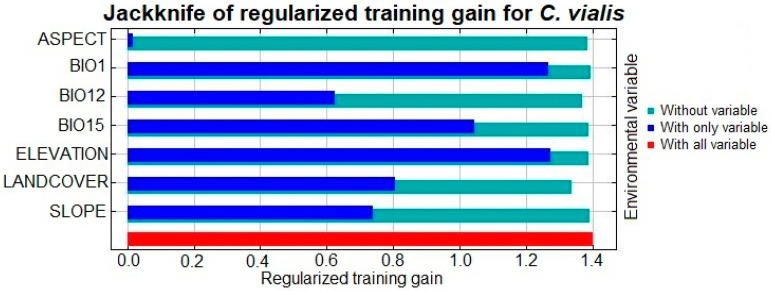
Analysis of selected environmental variables, i.e., aspect, BIO1, BIO12, BIO15, elevation, landcover, and slope, based on jackknife tests. Dark blue, light blue, and red bars interpret models on account of specific variables, in the absence of specific variables, and maximal performance.

To highlight the relative responses of these variables in the distribution and potential spread of *C. vialis*, an additional jackknife test was performed, which revealed that BIO1 and elevation produced the highest gain (or the maximum contribution), followed by BIO15, landcover, slope, and BIO12, whereas aspects showed nearly zero contribution (Figure 5).

**Figure 6:**
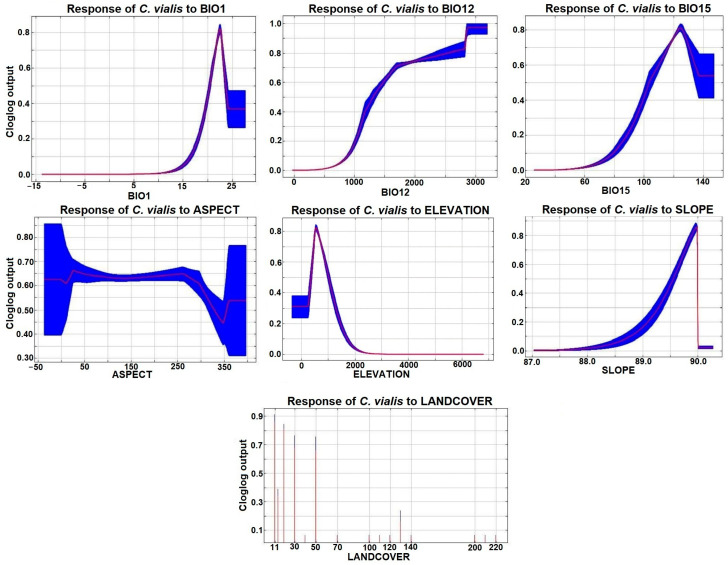
Response of *Calyptocarpus vialis* to selected environmental variables (aspect, BIO1, BIO12, BIO15, elevation, slope, and landcover) in terms of cloglog output. The mean response of 10 replicate MaxENT runs is depicted in red on the curve, while the mean ± 1 SD is presented in blue color. Two shades represent the categorical variable (landcover).

Further, the cloglog output was presented in the form of response curves, which depict changes in the logistic prediction in response to a particular environmental variable, with the remaining variables being constant at a mean sample value. According to the response curves, BIO1 and BIO12 predicted the presence of *C. vialis* within the ranges of 20–25 and 1300–3000, respectively (Figure 6). The response curve of BIO15 was the maximum within a range of 102–140 (Figure 6). Continuous data for aspect, elevation, and slope had peak response values in the range of −50 to 310, 300 to 1000, and 89.6 to 89.9, respectively (Figure 6). Elevation data showed the distribution of *C. vialis* up to 2000 m asl, with the maximum presence around 600–700 m asl (Figure 6). Along with bioclimatic variables, Globcover landcover categorical data showed that category 11 is the most important in forecasting the distribution of *C. vialis*, followed by categories 20, 30, and 50 (Figure 6).

### 3.3. Distribution of Calyptocarpus vialis

**Figure 7:**
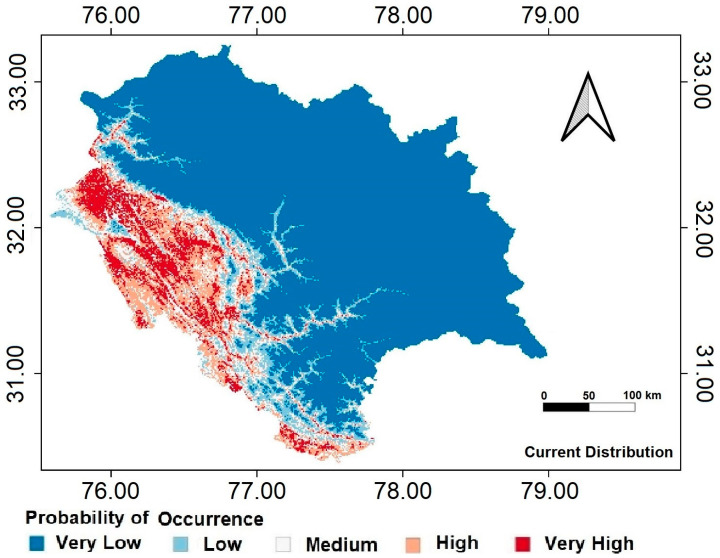
The current distribution of *Calyptocarpus vialis* in the study area based on MaxEnt modeling.

**Figure 8:**
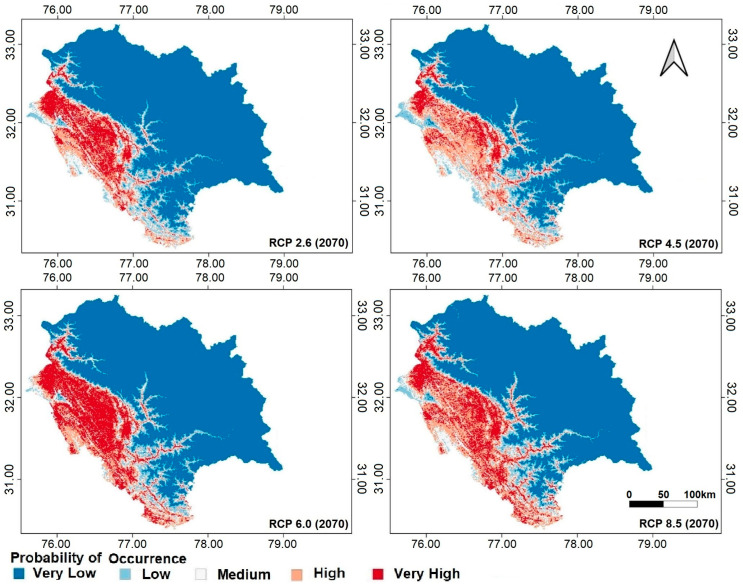
The probable niche distribution of *Calyptocarpus vialis* in Himachal Pradesh under future climatic scenarios (RCP 2.6, 4.5, 6.0, and 8.5) by 2070.

The current study describes the basic niche range of *C. vialis* in the studied area (Figure 7; Table 2). According to the model, nearly 14.17% of the Himachal Pradesh area is a “high” suitability area for *C. vialis* invasion (Figure 7). The “high” and “very high” suitability habitats are mostly found in the lower elevations, i.e., Una, Kangra, Hamirpur, Sirmour, Bilaspur, Mandi, and Chamba districts of Himachal Pradesh (Figure 7). On the other hand, the areas of high elevation have not been found suitable for *C. vialis* as per the model. Different RCP scenarios (2.6, 4.5, 6.0, and 8.5) predicted an expansion of suitable habitats for *C. vialis* and a gradual but consistent decline in “very low” suitability areas has been observed under different RCP scenarios by 2070 (Figure 8; Table 2).

According to the model, “high” suitability areas may expand to the maximum under RCP 8.5, while the same would be achieved by “very high” suitability areas under RCP 6.0 (Figure 8; Table 2). The model predicted that the emission route of RCP 2.6 will expand the potential future distribution area of *C. vialis* in Himachal Pradesh, with 8.64 and 8.46% of the area showing “high” and “very high” suitability for invasion, respectively (Figure 8; Table 2). According to the model, under RCP 4.5, the 11.77% area will be of “high” suitability, while only the 5.39% area will be denoted as “very high” suitable, thereby indicating that some suitability areas expanded while others contracted in this climate scenario (Figure 8; Table 2). Likewise, the RCP 6.0 scenario indicated that 9.13 and 13.19% area will transform into “high” and “very high” suitability areas, respectively, for *C. vialis* in comparison to the current situation, further confirming the possibility of enhancement of its niche (Figure 8; Table 2). Furthermore, a similar comparison with RCP 8.5 also indicates that 13.07 and 10.59% of the area will transform into “high” and “very high” suitability areas, respectively, for *C. vialis* as per the model (Figure 8; Table 2). Both RCP 6.0 and 8.5 demonstrate the habitat expansion of *C. vialis* by 2070 (Figure 8; Table 2).

According to the model, the middle northwestern Himalayan region is likely to become more suitable for *C. vialis* in all the scenarios, and the overall current distribution (with habitat suitability ranging from “medium” to “very high”) will be maintained. The model predicted that the total vulnerable area (with habitat suitability from “medium” to “very high”) in 2070 with RCP scenarios 6.0 and 8.5 will be 28.76 and 31.68%, respectively (Figure 8; Table 2). This is approximately 7 and 10% greater than the habitat suitability area of *C. vialis* (“medium” to “very high”), respectively, under the current distribution scenario (21.59%) (Figure 8; Table 2).

## 4. Discussion

Human-driven climate change has a direct impact on vegetation patterns, particularly the distribution of non-native species [54]. This is especially true for biodiversity hotspots, which have relatively fragile ecosystems and a high level of endemism [55]. The Himalayas are one such unique, ecologically significant, and biodiverse ecoregion that is recognized globally for its diverse endemic vegetation. It is important to comprehend the potential distribution and habitats susceptible to invasive species in this ecoregion to facilitate the ecosystem conservation efforts [16]. *C. vialis* is an alien species invading the lower elevations of the northwestern Himalayas [29]. This study is the first empirical investigation of the fundamental niche range of *C. vialis* in the northwestern IHR, and how it changes in response to future environmental scenarios.

ENM is exploited as a measure to evaluate the present and future spread of invasive taxa with respect to environmental parameters [56]. MaxEnt was used in the present study, which has been put into practice for several rare, threatened, and invasive exotic species [16,21,22,57,58]. The model was found to fit the criteria commonly used to predict the accuracy of the model [59,60]. Since the model can predict the future invasion dynamics besides the current potential distribution under different RCP scenarios, it represents multiple possibilities of niche contraction and expansion according to the interactions among environmental variables.

### 4.1. Significance of Predictor Variables

The study revealed that among all the predictor variables, BIO15, i.e., precipitation seasonality (38.8%), and BIO1, i.e., annual mean temperature (35.7%), have the most significant impact on the probable distribution flux of *C. vialis.* Likewise, several other reports suggest a significant role of temperature and precipitation-based variables in species’ dispersal in alien environments [61,62,63]. Both climatic variables, i.e., temperature and rainfall, affect plant development and metabolism, such as physiology, reproductive potential, and dispersal strategies, thereby interfering with the adaptive survival and habitat range of the species [15,64].

Additionally, topographic variables like elevation (11.7%) and landcover (6.3%) were also found to impact the habitat suitability of *C. vialis* as per the model. By restricting the distribution of *C. vialis* to an elevation range of <2000 m via a response curve, the model demonstrated that the spread of *C. vialis* is significantly regulated by elevation. Studies have confirmed an intense impact of elevation on the potential spread of invasives as mountainous regions act as a physical and environmental barrier, delimiting their survival and dispersal [42,65]. The current findings also validate an earlier study that reported its distribution in the lower Himalayan regions [35]. On the other hand, landcover is a critical variable that is frequently associated with the prevalence of a species [66,67]. Disturbance-induced changes in the landcover have already been recognized as a pivotal driver for the entry and establishment of alien species in non-native ranges [68]. The results of the present study extend support to the hypothesis that potential natural and/or anthropogenically induced disturbances and land use changes that affect the landcover may also play a key role in shaping the niche of *C. vialis*.

### 4.2. Expansion of Suitable Habitats for C. vialis under Future Climate Scenario

ENM revealed the possibility of contraction as well as expansion in the habitats of *C. vialis* by 2070 under different RCP scenarios. With a few exceptions, results clearly show contraction in areas that were “very less” or “less” suitable for the species and expansion in areas of “high” and “very high” suitability for the species when compared to the current scenario. Habitat expansion in “high” and “very high” suitable regions might be due to the creation of a more conducive environment resulting from a pronounced climate change trajectory in the form of elevated temperatures and altered precipitation patterns. Similar results showing a climate-induced expansion of suitable habitats for various invasive species by 2070 have been reported [25,62,69,70,71,72,73]. On the contrary, contraction in “very high” suitable habitat under RCP scenario 4.5 might be due to a combination of factors such as stabilizing radiative forcing and moderate changes in temperature and precipitation, which can alter species-specific responses [74].

The potential current distribution of *C. vialis* represents 21.59% of the state’s area with “medium” to “high” invasion suitability; however, under the RCP 8.5 scenario, it is likely to expand by 10% by 2070. This implies that the habitats which are currently unsuitable or less suitable for *C. vialis* might evolve into suitable habitats in the near future. The findings, therefore, indicate that changes in climatic patterns will hasten the spread and abundance of alien invaders in the near future [65]. Rising temperatures can be speculated to be one of the most beneficial aspects of climate change for alien species [16]. Since the plasticity or variability in plants towards warming in terms of phenotypic changes, functional traits, and phenological responses is supposed to determine their dominance in the upcoming decades [75,76,77], *C. vialis* is an appropriate candidate to meet the criteria since it is an *r*-selected species with an extended, fast, and fecund life cycle and ability to adapt to warm conditions. Many weeds have been reported to expand their range due to climate change as they can readily adapt using their invasive capabilities such as phenotypic plasticity, fast growth, and wide ecological adaptability [51,78,79].

### 4.3. Niche expansion of C. vialis from Lower to Higher Elevations

At present, *C. vialis* is established at lower elevations with only a scattered presence at elevations above 1500 m asl; however, the current study indicates a “high” probability of niche expansion of *C. vialis* at both lower and higher elevations. Temporal niche expansion along an elevational gradient is commonly noticed in invasive species [80], which is usually caused by changes in micro-climatic conditions [80,81]. It can be further manifested by species’ broad adaptability [29] and anthropogenic disturbances in the study area due to the growth of the trade and tourism sectors [82]. Characteristics of invasive species such as wide ecological amplitude, adaptive evolution, and phenotypic plasticity also play a critical role in the altitudinal expansion [12,83]. Many invasive species have been reported to expand their niche from plains to lower altitudinal regions and from lower altitudinal regions to higher altitudinal regions [12,84].

### 4.4. Management of C. vialis in the Northwestern IHR Demands Meticulous Attention

With niche expansion, the negative effects of invasive species on the ecosystem and community worsen [83]. Therefore, identifying and prioritizing the management of invasive species-prone locations is pertinent for policymakers, conservationists, and ecologists to limit their potential ecological and socio-economic impacts. ENM, as a predictor of invasion dynamics, is a simple and low-cost technique for forecasting invasion-prone regions, deploying timely monitoring and efficient response systems, and designing suitable approaches to check potential invasions [59]. The current investigation implies that to restrict the further spread of *C. vialis* in the study region, scientifically informed management policies should be created and implemented, taking into consideration the ongoing and potential changes in temperature, precipitation, and landcover in the study area. Strong quarantine measures and regular monitoring are advised for the potential hotspots identified via ENM, especially those that have not yet been invaded by *C. vialis*. Further ground validation of the results reported in the present study via field investigations and a long-term monitoring approach is also crucial. Additionally, estimating the ecological and socio-economic impacts of *C. vialis* at spatio-temporal scales may also provide more thorough insights into the invasion dynamics of the species.

## 5. Conclusions

Using the MaxEnt modeling approach, we predicted the potential invasion of *C. vialis* in the northwestern IHR under a climate change scenario. Future invasion by *C. vialis* was found to be influenced by bioclimatic and topographic variables, particularly precipitation seasonality, annual mean temperature, elevation, and landcover. The current distribution of *C. vialis* represents 21.59% of the state with “moderate” to “high” invasion suitability; however, under the RCP 8.5 scenario, it is likely to expand by 10% by 2070. Also, the present study indicates a “high” probability of niche expansion for *C. vialis* at both lower and higher elevations. These findings can be used by policymakers and land-use managers to monitor potential hotspots of *C. vialis* and implement scientifically sound strategies for the management of the species.

## Figures and Tables

**Table 1:** Bioclimatic and topographic parameters (along with their contribution) retained after the Pearson multicollinearity test used for predicting the suitable habitats of *Calyptocarpus vialis*.

Predictor Variables (Codes; Unit)	Percent Contribution	Permutation Importance
Annual mean temperature (BIO1; °C)	35.7	57.1
Annual precipitation (BIO12; mm)	1.6	4.7
Precipitation seasonality (BIO15)	38.8	1.9
Landcover	6.3	4.9
Elevation (m; asl)	11.7	29.5
Slope gradient (slope; degree)	5	1.1
Slope aspect (aspect; degree)	1	0.7

**Table 2:** Habitat suitability for *Calyptocarpus vialis* in Himachal Pradesh under current and future climatic scenarios by 2070.

Climatic Scenario		Habitat Suitability				
		Very Low	Low	Medium	High	Very High
Current	Area (km^2^)	46,129.26	5330.18	4871.96	5605.29	3693.30
	Percent of total study area (%)	70.29	8.12	7.42	8.54	5.63
RCP 2.6	Area (km^2^)	43,713.49	5457.42	5240.77	5667.18	5551.12
	Percent of total study area (%)	66.61	8.32	7.99	8.64	8.46
RCP 4.5	Area (km^2^)	42,007.83	6077.26	6281.87	7723.60	3539.41
	Percent of total study area (%)	64.01	9.26	9.57	11.77	5.39
RCP 6.0	Area (km^2^)	41,902.95	4853.04	4225.46	5991.29	8657.24
	Percent of total study area (%)	63.85	7.39	6.44	9.13	13.19
RCP 8.5	Area (km^2^)	39,714.13	5123.85	5262.26	8576.43	6953.30
	Percent of total study area (%)	60.51	7.81	8.02	13.07	10.59

## Data Availability

The data presented in this study are available on request from the corresponding author.
